# Maladaptive Daydreaming in an Adult Italian Population During the COVID-19 Lockdown

**DOI:** 10.3389/fpsyg.2021.631979

**Published:** 2021-03-24

**Authors:** Alessandro Musetti, Christian Franceschini, Luca Pingani, Maria Francesca Freda, Emanuela Saita, Elena Vegni, Corrado Zenesini, Maria Catena Quattropani, Vittorio Lenzo, Giorgia Margherita, Daniela Lemmo, Paola Corsano, Lidia Borghi, Roberto Cattivelli, Giuseppe Plazzi, Gianluca Castelnuovo, Eli Somer, Adriano Schimmenti

**Affiliations:** ^1^Department of Humanities, Social Sciences and Cultural Industries, University of Parma, Parma, Italy; ^2^Department of Medicine and Surgery, University of Parma, Parma, Italy; ^3^Department of Biomedical, Metabolic and Neural Sciences, University of Modena and Reggio Emilia, Modena, Italy; ^4^Department of Humanities, University of Naples Federico II, Naples, Italy; ^5^Department of Psychology, Catholic University of the Sacred Heart, Milan, Italy; ^6^Department of Health Sciences, Faculty of Medicine and Surgery, University of Milan, Milan, Italy; ^7^IRCCS Institute of Neurological Sciences of Bologna (ISNB), Bologna, Italy; ^8^Department of Clinical and Experimental Medicine, University of Messina, Messina, Italy; ^9^Dipartimento di Scienze della Società e della Formazione d'Area Mediterranea, Università per Stranieri Dante Alighieri, Reggio Calabria, Italy; ^10^Istituto Auxologico Italiano IRCCS, Psychology Research Laboratory, Milan, Italy; ^11^Faculty of Social Welfare and Health Sciences, School of Social Work, University of Haifa, Haifa, Israel; ^12^Faculty of Human and Society Sciences, Kore University of Enna, Enna, Italy

**Keywords:** maladaptive daydreaming, depression, anxiety, stress, COVID-19

## Abstract

During the COVID-19 outbreak, individuals with or without mental disorders may resort to dysfunctional psychological strategies that could trigger or heighten their emotional distress. The current study aims to explore the links between maladaptive daydreaming (MD, i.e., a compulsive fantasy activity associated with distress and psychological impairment), psychological symptoms of depression, anxiety, and negative stress, and COVID-19-related variables, such as changes in face-to-face and online relationships, during the COVID-19 lockdown in Italy. A total of 6,277 Italian adults completed an online survey, including socio-demographic variables, COVID-19 related information, the 16-item Maladaptive Daydreaming Scale (MDS-16), and Depression, Anxiety, and Stress Scales-21 Items (DASS-21). Based on an empirically derived cut-off score, 1,082 participants (17.2%) were identified as probable maladaptive daydreamers (MDers). A binary logistic regression revealed that compared to controls, probable MDers reported that during the COVID-19 lockdown they experienced higher levels of anxiety and depression, decreased online social relationships, and, surprisingly, stable or increased face-to-face social relationships. Given the peculiar characteristics of the pandemic context, these findings suggest that the exposure to the risk of contagion had probably exacerbated the tendency of probable MDers to lock themselves inside their mental fantasy worlds, which in turn may have contributed to further estrangement from online social relationships and support, thus worsening their emotional distress.

## Introduction

The new coronavirus SARS-CoV-2 and its related syndrome, named COVID-19 by the World Health Organization (WHO), has evolved into a global health threat. In the early months of 2020, the infection showed extreme virulence (She et al., 2020), rapidly spreading from the city of Wuhan to most countries in the world. Subsequently, Italy became one of the major COVID-19 hotspots. On March 9th, 2020, the Italian Prime Minister announced a government lockdown decree featuring the slogan “I stay at home” (Italian Ministry of Health, 2020). The new regulations employed pandemic control policies based on social distancing aimed to minimize contacts with potentially infected individuals. However, while domestic lockdown may have helped to control the physical health emergency (Muggeo et al., 2020), the experience of forced isolation severely impaired people's social and economic well-being, resulting in a negative mental health impact (Marazziti, 2020) because of increased loneliness and anxiety (Schimmenti et al., 2020a). As described by Brooks et al. (2020), the psychological impact of prolonged quarantine included post-traumatic stress symptoms, confusion, and anger determined by the duration of lockdown, fear of infection, feelings of frustration and tedium, inadequate availability of supplies, inconsistent information, financial loss, and stigma.

Although the psychological impact of COVID-19 is not yet fully understood, the available empirical literature has provided some important clues. For instance, a prevalence of moderate to severe depression, anxiety, and stress levels ranging from 8.1% (for stress) to 28.8% (for anxiety) (Wang et al., 2020a), and no decrease in psychological symptoms 4 weeks after the initial outbreak (Wang et al., 2020b) were found in the Chinese population. Similar results were found in the Italian context, showing high levels of psychological distress experienced in the Italian population during the COVID-19 outbreak (Colizzi et al., 2020; Favieri et al., 2020; Mazza et al., 2020; Moccia et al., 2020; Schimmenti et al., 2020b). Moreover, a large-sample cross-sectional study by Rossi et al. (2020) on 18,147 individuals showed a high prevalence of negative mental health outcomes in the general Italian population, with elevated rates of post-traumatic stress symptoms (37%) (17.3%), anxiety (20.8%), insomnia (7.3%), high perceived stress (21.8%), and adjustment disorders (22.9%). However, it is important to consider that in the first phase of the COVID-19 outbreak (the most critical phase of this pandemic), the restrictive measures adopted in Italy were extreme and unprecedented, unlike other European and non-European countries. Specifically, the PsyCOVID longitudinal study by Cerami et al. (2020) showed that individuals living in Northern Italy—the area most affected by the COVID-19 epidemic in the whole of Europe—reported more detrimental effects on health due to the outbreak than individuals living in the Central and Southern regions. Furthermore, as highlighted by the same authors, increased levels of distress and loneliness associated with social isolation and the profound destabilization of life, may exacerbate the risk of mental health problems, even in the general population. It is plausible, therefore, that under the threat of a highly contagious and untreatable disease, maladaptive psychological strategies may develop in healthy individuals, as well as aggravate the pre-existing psychiatric conditions (Mucci et al., 2020). For example, recent evidence has shown that to ease COVID-19-related distress individuals were more prone to using psychoactive substances and engage in potentially addictive behaviors, such as social networking, surfing the Internet, and gaming (King et al., 2020; Mestre-Bach et al., 2020). Furthermore, Seçer and Ulaş (2020) showed that COVID-19-related avoidance responses, such as distraction or denial, may play a pivotal role in the development and maintenance of negative psychological outcomes. People in quarantine may also be more prone to employing mental escapism in response to a distressful external reality, by becoming absorbed in their inner worlds (Mucci et al., 2020).

A growing body of literature deals with a newly emerging absorption disorder, known as maladaptive daydreaming (MD) and conceptualized as a dysfunctional form of imaginative involvement, defined as “extensive fantasy activity that replaces human interaction and/or interferes with academic, interpersonal, or vocational functioning” (Somer, 2002, p. 199). In the first seminal work on six maladaptive daydreamers (MDers) (Somer, 2002), the central MD themes included a description of captivity, rescue and escape, and idealized self. MDers can spend hours completely absorbed in vivid and highly structured fantasies experiencing a high sense of presence in the daydream (Somer et al., 2016a), often engaging in stereotypical movements, such as swinging, or pacing to facilitate their absorption in fantasy (Bigelsen and Schupak, 2011; Somer et al., 2016b). Although daydreaming is a widespread (Singer, 1966; Klinger, 1990) and normal mental experience (Killingsworth and Gilbert, 2010; Bigelsen et al., 2016), MD is a clinical phenomenon in which an individual is extensively, often compulsively, absorbed in an internal fantasy world that is associated with impairment in a variety of important areas of functioning (Somer et al., 2017a).

Given the association with adverse childhood experiences, Somer initially theorized that MD is a coping strategy gone awry, originally aimed at attenuating feelings of emotional pain and loneliness, and mentally escaping from adverse environments (2002; Somer and Herscu, 2017). Although daydreaming could be a pleasant activity, as a coping strategy it is dysfunctional because it can generate a vicious cycle of social isolation and distress, which in turn may further increase the need to self-soothe by daydreaming (Bigelsen and Schupak, 2011). Growing evidence indicates that MD is a valid, reliable, and distinct clinical construct characterized by repeated unsuccessful efforts to control fantasy activity, intense shame, and exhaustive efforts to conceal this behavior, which leads to impairment in social, family, and work-related activities (Somer et al., 2017b). Since these clinical features resemble those observed in addictive behaviors, MD has been nosographically framed by some authors as a behavioral addiction (Somer et al., 2016b; Pietkiewicz et al., 2018; Schimmenti et al., 2020c; Soffer-Dudek et al., 2020).

The abnormality of MD is evident by its comorbidity with other psychiatric conditions and with global psychopathology (Bigelsen et al., 2016; Somer and Herscu, 2017; Somer et al., 2017a; Soffer-Dudek and Somer, 2018; Schimmenti et al., 2020c). The most frequent comorbid DSM-5 disorders are attention-deficit hyperactivity disorder, anxiety disorders, depressive disorder, obsessive-compulsive or related disorder (Somer et al., 2017a). Specifically, many MDers with anxiety and depression were more likely to engage in MD as a means to flee from their unpleasant circumstances (Somer, 2002; Alenizi et al., 2020). Conversely, comorbidity with psychosis was rare. Despite the serious clinical manifestations of MD, reality testing among MDers remained intact as they reported an intact ability to distinguish between fantasy and reality (Bigelsen and Schupak, 2011; Schimmenti et al., 2019).

As for etiopathogenesis, Soffer-Dudek and Somer (2018) proposed a stress-diathesis model for MD, in which individuals who have an innate predisposition to immerse themselves in an internal fantasy world may become MDers if they are exposed to stressful or traumatic life events. In line with this perspective, several studies have suggested that individuals are likely to take shelter in comforting daydreams in the context of stressful circumstances and mental pain (e.g., Greenwald and Harder, 1994, 2003). Importantly, beyond traumatic life events, current social isolation has also been indicated as one of the most relevant factors affecting the development and maintenance of MD (Somer et al., 2016b; Somer and Herscu, 2017). Again, a circular dynamic is triggered: MDers frequently report childhood aloneness as a prelude to their immersion in their compensatory inner world, which in turn exacerbates their isolation from the real social world (Somer et al., 2016b). Hence, considering that social isolation might represent an important risk factor for MD, the COVID-19 lockdown is an unprecedented model with which to examine the interrelationships between MD and psychopathological symptoms in real-time.

The current report aims to explore the relationships between MD, psychological symptoms (depression, anxiety, and negative stress), and COVID-19-related variables (e.g., changes in face-to-face and online relationships) in a large sample of Italian adults from 10th March to 4th May 2020, the first COVID-19 lockdown period in Italy employed during an unprecedented mass disaster. In view of the reviewed literature, we expected a pattern of positive associations between decreased face-to-face and online social relationships, and MD as well as between psychological symptom levels and MD.

## Materials and Methods

### Participants

We circulated a call for research participants in several Italian universities. We surveyed an online convenience sample of 6,277 participants (1,685 males, 26.8%; 4,592 females, 73.2%) aged from 18 to 82 years (*M* = 33.62 years, *SD* = 13.46). All the questions were mandatory, and so there were no missing cases. The socio-demographic characteristics of the sample are described in Tables 1A,B.

**Table 1:** Socio-demographic characteristics the participants.

**A**	
	*N* = 6,277
**Demographic data**	
***Gender, n (%)***	
Males	1,685 (26.8)
Females	4,592 (73.2)
***Age (years old), n (%)***	
18–25	2,538 (40.4)
26–30	1,019 (16.2)
31–40	902 (14.4)
41–50	771 (12.3)
51–60	806 (12.8)
>60	241 (3.8)
***Education level, n (%)***	
Elementary/Middle school	213 (3.4)
High school	2,948 (47.0)
Bachelor's degree	1,191 (19.0)
Master's degree	1,418 (22.6)
Doctoral degree	507 (8.1)
***Marital status, n (%)***	
Single	2,192 (34.9)
Married or re-married	1,581 (25.2)
Cohabitant	577 (9.2)
In a relationship	1,645 (26.2)
Divorced/separated/widowed	282 (4.5)
***Children (yes), n (%)***	
Yes	1,797 (28.6)
No	4,480 (71.4)
***Number of people with whom the participant lived with during the lockdown, n (%)***	
0	461 (7.3)
1	1,345 (21.4)
2	1,531 (24.4)
3	1,885 (30.0)
4	802 (12.8)
5+	253 (4.0)
***Occupation, n (%)***	
Retired	114 (1.8)
Student	1,803 (28.7)
Working student	830 (13.2)
Healthcare employee (public/private)	360 (5.7)
Police/military	52 (0.8)
Artisan, laborer, farmer	100 (1.6)
Employee/manager/owner of business activity	587 (9.4)
Employee/manager/owner of industrial activity	394 (6.3)
Intellectual profession	521 (8.3)
Unemployed/searching	287 (4.6)
Office executive job	32 (0.5)
Technical profession	317 (5.1)
Unskilled job	776 (12.4)
Other	104 (1.6)
***Job loss during the lockdown***	
Yes	2,963 (47.2)
No	3,314 (52.8)
***Work in direct contact with the public during the lockdown***	
Yes	3,993 (63.6)
No	2,284 (36.4)
***Residence area***	
North	4,239 (65.5)
Centre	457 (7.3)
South	1,581 (25.2)
**B**	
	*N* = 6,277
**COVID-19 related data**	
***COVID-19 positive, n (%)***	
No	6,029 (96.0)
Yes	48 (0.8)
Had symptoms but no swab test	88 (1.4)
No answer/other	85 (1.4)
***Forced quarantine, n (%)***	
No	5,725 (91.2)
Yes	532 (8.5)
No answer	20 (0.3)
***Someone close positive, n (%)***	
Yes	924 (14.7)
No	5,353 (85.3)
***Someone close died, n (%)***	
Yes	412 (6.6)
No	5,865 (93.4)
***Changes in face-to-face relationships, n (%)***	
Decreased	5,526 (88.0)
Stable	347 (5.5)
Increased	404 (6.4)
***Changes in online relationships, n (%)***	
Decreased	334 (5.3)
Stable	1,975 (31.5)
Increased	3,968 (63.2)

### Procedures

This study is part of a larger research project named “Resilience and the COVID-19: reacting to perceived stress. Effects on sleep quality and diurnal behavior/thoughts.” The first data from the larger survey were published elsewhere (Franceschini et al., 2020; Lenzo et al., 2020). Ethical clearance was obtained from the Ethics Committee of the Center for Research and Psychological Intervention (CERIP) of the University of Messina. The study adhered to the Ethical Code of the Italian Association of Psychology (AIP) and the American Psychological Association (APA). The inclusion criteria were being an adult (i.e., at least 18 years old), being an Italian speaker, and living in Italy during the COVID-19 lockdown. Participants provided informed consent and completed an anonymous questionnaire that addressed socio-demographic information, COVID-19-related data, maladaptive daydreaming, and psychopathological symptoms (depression, anxiety, and negative stress). Anonymity was guaranteed, as no data on the participants' identification, or their Internet Protocol address, were collected. Participants did not receive any fee for their involvement in the study.

### Measures

#### Socio-Demographics

To obtain a profile of the respondents' demographic features we asked about age, gender, education level, occupation, marital status, having children, number of family members, employment, house size, having a garden, and area of residence.

#### The Maladaptive Daydreaming Scale (MDS−16)

The 16-item Maladaptive Daydreaming Scale (MDS**–**16; Somer et al., 2016c; Italian version by Schimmenti et al., 2020c) was used to measure the degree of maladaptive daydreaming among participants. The Italian version of the MDS**–**16 includes two subscales: Interference with life (8 items, e.g., “Some people experience difficulties in controlling or limiting their daydreaming. How difficult has it been for you to keep your daydreaming under control?”) and Somato-sensory retreat (8 items, e.g., “Some people notice that certain music can trigger their daydreaming. To what extent does music activate your daydreaming?”; see Supplementary Table 1 for the questionnaire). Participants were asked to respond to each item on an 11-point Likert-type scale ranging from 0% (*never*/*none of the time*) to 100% (*extremely frequent*/*all of the time*), with 10% increments. There are no reversed items. Overall MDS-16 scores are the average of each item, with higher scores indicating higher levels of MD. Scores of 51 or above (Schimmenti et al., 2020c) have been used to discriminate between MDers and non-MDers with excellent sensitivity (90.37%). The MDS-16 showed excellent psychometric properties not only in the Italian version of the instrument (Schimmenti et al., 2020c), but also in the English (Somer et al., 2016c), Hebrew (Jopp et al., 2019), and Arabic (Abu-Rayya et al., 2020), and Hungarian versions (Sándor et al., 2020). In the present study, Cronbach's alpha was 0.92.

#### The Depression Anxiety Stress Scale-21 (DASS-21)

The short form of the Depression, Anxiety, and Stress Scale-21 Items (DASS-21—Lovibond and Lovibond, 1995; Italian version by Bottesi et al., 2015) was used to assess the psychological symptoms among participants. The DASS-21 is a self-report tool in which participants rate the frequency and the severity of depression (e.g., “I felt that life was meaningless”), anxiety (e.g., “I felt I was close to panic”), and negative stress (e.g., “I found it hard to relax”) for the previous week. Each of the three DASS-21 scales includes seven items, where each item is ranged on a 4-point scale (0 = “Did not apply to me at all,” to 3 = “Applied to me very much, or most of the time”). Subscale total scores are multiplied by 2 to suit the original version of the DASS and ranged from 0 to 42, with higher scores indicating a more severe level of depression, anxiety, and negative stress. The cut-off values for severe depression, anxiety, and negative stress were ≥21, ≥15, and ≥26, respectively (Lovibond and Lovibond, 1995). The Cronbach's α values for each subscale in this study were 0.89 (depression), 0.83 (anxiety), and 0.91 (negative stress), respectively.

### COVID-19 Lockdown Related Information

The following variables related to the COVID-19 outbreak were investigated: COVID-19 diagnosis (yes, no, had symptoms but no swab test), forced quarantine (yes or no), someone close was positive for COVID-19 (yes or no), mourning related to COVID-19 (yes or no), face-to-face and online social relationship changes (decreased, stable, increased).

### Data Analyses

Descriptive statistics were calculated for all the study variables. A multi-categorical logistic regression analysis was used to define possible predictors of MD. We employed the Hosmer and Lemeshow Test to verify whether the model fits the data. The dependent variable was obtained by dichotomizing MDers and non-MDers via the MDS-16 cut-off value of 51 to identify positive cases (see Schimmenti et al., 2020c). Independent variables were gender, age, education, residence area, having children, marital status, job loss during the lockdown, working in direct contact with the public during the lockdown, having been infected by the coronavirus, having been in quarantine, having someone close infected by the coronavirus, loss of a loved one due to the pandemic, number of people with whom the participant was living with during the lockdown, house size (in square meters) of the location in which the respondent was living during the lockdown, the availability of a garden in that location, perceived changes in the frequency of the respondent's face-to-face and online relationships, negative stress, anxiety and depression levels as measured by DASS-21 variables.

## Results

Of the total sample of 6,277 participants, 1,082 (17.2%) reported clinical levels of MD (MDS-16 mean score > 50) and were identified as self-reported MDers. The logistic regression model was statistically significant (χ^2^ = 569.35; df = 40; *p* < 0.001) while the Hosmer and Lemeshow Test was not significant (χ^2^ = 10.606; df = 48; *p* = 0.23): thus, the model fits the data and could be further interpreted. The model explained 15.2% of pseudovariance (Nagelkerke *R*^2^) and correctly classified 82.60% of cases. As Table 2 shows, MD was not associated with gender (*p* = 0.14) and was negatively associated with two categories of marital status: being in a romantic relationship (*p* = 0.02; OR: 0.70; 95% CI: 0.52–0.95) and non-marital cohabitation with the partner (*p* > 0.001; OR: 0.67; 95% CI: 0.56–0.79). MDers were less likely to have a doctorate or a professional diploma (*p* = 0.002; OR: 0.44; 95% CI: 0.27–0.74). Furthermore, age was negatively associated with MD (*p* > 0.001; OR: 0.98; 95% CI: 0.97–0.99). While negative stress was not associated with MD, we found that MD was significantly correlated at a *p* < 0.001 level with mild (OR: 1.59; 95% CI: 1.23–2.09), moderate (OR: 1.75; 95% CI: 1.42–2.16), severe (OR: 1.68; 95% CI: 1.26– 2.25), or extremely severe (OR: 2.60; 95% CI: 2.00–3.48) anxiety. Our data also show that MD was significantly linked with mild (OR: 1.90; 95% CI: 1.52–2.37), moderate (OR: 2.18; 95% CI: 1.74–2.74), severe (OR: 2.87; 95% CI: 2.16– 3.83), or extremely severe (OR: 3.23; 95% CI: 2.35–4.43) depression. Furthermore, MD was associated with stable (*p* < 0.001; OR: 1.84; 95%, CI: 1.39–2.43) and elevated (*p* = 0.045; OR: 1.31; 95%, CI: 1.01– 1.69) frequencies of face-to-face relationships. In contrast, MD was negatively associated with stable (*p* = 0.001; OR: 0.59, 95% CI: 0.44–0.80) and elevated (*p* = 0.02; OR: 0.70, 95% CI: 0.52–0.94) frequencies of online relationships. The number of people in the respondents' households during the lockdown and the characteristics of their work did not predict MD.

**Table 2:** Multivariable logistic regression analysis of the probable MDers sample.

		**Estimate**	**E.S**.	**Wald**	**gl**	***p***	**OR**	**95% C. I**.	
								**Lower**	**Upper**
**Demographic data**									
*Male gender*		−0.13	0.09	2.17	1	0.14	0.88	0.74	1.04
Level of education	Elementary/middle school			11,51	4	0.02			
	High school graduation	−0.26	0.19	1.75	1	0.19	0.78	0.53	1.13
	Bachelor's degree	−0.23	0.20	1.31	1	0.25	0.79	0.53	1.18
	Master's or specialist degree	−0.32	0.20	2.43	1	0,12	0.73	0.49	1.09
	Doctorate or graduate school	−0.81	0.26	9,87	1	0.002	0.44	0.27	0.74
Residence area	North			4.48	2	0,11			
	Centre	0.27	0.14	3.88	1	0.05	1.31	1.00	1.71
	South	0.10	0.09	1.32	1	0.25	1.10	0.93	1.31
*Having children*		0.20	0.17	1.43	1	0.23	1.22	0.88	1.69
Marital status	Single			23.66	4	>0.001			
	Married or re-married	−0.29	0.17	2.87	1	0.09	0.75	0.54	1.05
	In a sentimental relationship	−0.35	0.15	5.34	1	0.02	0.70	0.52	0.95
	Living with the partner but not married	−0.41	0.09	21.01	1	<0.001	0.67	0.56	0.79
	Divorced or Separated or Widowed	−0.10	0.24	0.16	1	0.69	0.91	0.56	1.46
*Age*		−0.19	0.01	13.26	1	<0.001	0.98	0.97	0.99
		**Estimate**	**E.S**.	**Wald**	**gl**	***p***	**OR**	**95% C. I**.	
								**Lower**	**Upper**
**Housing condition**									
Square meters of the house where he/she spent the lockdown		≤80			4.68	3	0.20		
	81–100	−0.10	0.11	0.79	1	0.37	0.91	0.74	1.12
	101–150	0.12	0.11	1.27	1	0.26	1.3	0.92	1.38
	>150	−0.02	0.11	0.03	1	0.86	0.98	0.79	1.23
Number of people with whom the participant lived with during the lockdown		0.01	0.03	0.09	5	0.77	1.01	0.95	1.07
The respondent's house has a garden		0.05	0.13	0.15	1	0.69	1.05	0.82	1.35
**Professional condition**									
Job loss during the lockdown		−0.04	0.08	0.29	1	0.60	0.96	0.83	1.11
Work in direct contact with the public during the lockdown		0.03	0.08	0.16	1	0.69	1.03	0.89	1.20
		Estimate	E.S.	Wald	gl	*p*	OR	95% C. I.	
								Lower	Upper
**COVID-19 related data**									
The respondent has lost loved ones		−0.11	0.16	0.46	1	0.50	0.90	0.66	1.23
The respondent was in quarantine		0.21	0.13	2.90	1	0.09	1.24	0.97	1.58
The respondent was infected with the coronavirus		0.38	0.39	0.95	1	0.33	1.47	0.68	3.16
The respondent had someone close infected		−0.01	0.11	0.02	1	0.90	0.99	0.80	1.22
Changes in the frequency of the face-to-face relationship	Decreased			20.97	2	<0.001			
	Stable	0.61	0.14	18.26	1	<0.001	1.84	1.39	2.43
	Increased	0.27	0.13	4.02	1	0.045	1.31	1.01	1.69
Changes in the frequency of online relationship	Decreased			12.45	2	0.002			
	Stable	−0.53	0.16	11.53	1	0.001	0.59	0.44	0.80
	Increased	−0.36	0.15	5.83	1	0.02	0.70	0.52	0.94
**Mental health data**									
Negative stress	Normal			5.27	4	0.26			
	Mild	0.17	0.12	1.85	1	0.17	1.18	0.93	1.50
	Moderate	−0.13	0.12	1.17	1	0.30	0.88	0.69	1.11
	Severe	−0.04	0.14	0.07	1	0.79	0.96	0.73	1.27
	Extremely severe	0.002	0.19	0.001	1	0.99	1.00	0.70	1.44
Anxiety	Normal			56.08	4	<0.001			
	Mild	0.47	0.14	12.13	1	<0.001	1.59	1.23	2.09
	Moderate	0.56	0.11	27.67	1	<0.001	1.75	1.42	2.16
	Severe	0.52	0.15	12.44	1	<0.001	1.68	1.26	2.25
	Extremely severe	0.97	0.14	46.93	1	<0.001	2.60	2.00	3.48
Depression	Normal			75.21	4	<0.001			
	Mild	0.64	0.11	31.53	1	<0.001	1.90	1.52	2.37
	Moderate	0.78	0.12	46.07	1	<0.001	2.18	1.74	2.74
	Severe	1.06	0.15	51.93	1	<0.001	2.87	2.16	3.83
	Extremely severe	1.17	0.16	52.48	1	<0.001	3.23	2.35	4.43

## Discussion

The current study aimed to explore the associations between contextual factors related to the COVID-19 lockdown, mental health variables, and MD in a large sample (*N* = 6,277) of Italian adults during the first COVID-19 lockdown period in Italy: 1,082 participants (17.2%) met the cut-off score for probable MD. This prevalence is quite high, considering that previous studies found similar incidence rates of MD in clinical groups (e.g., Somer et al., 2019a). Our data thus deserve some consideration. First, it has already been noted that public health emergencies, such as the COVID-19 outbreak, may deeply affect the well-being and mental health of individuals in the affected community (Pfefferbaum and North, 2020). Hence, this finding may be partially explained by the heightened levels of psychological symptoms already reported in the context of this global mass disaster and reflect a general peri-traumatic deterioration in mental health. Further caution should be employed when interpreting our results because we cannot claim universality for our findings.

The demographic data we collected were in line with previous studies that reported higher levels of MD among young adults (Zsila et al., 2019), no gender differences (although female participants are more represented among MDers; see Schimmenti et al., 2020c), low levels of MD in individuals with higher education (Somer et al., 2016c) and among those who are not in a romantic relationship (Somer et al., 2016b).

MDers endorsed higher levels of anxiety and depression symptoms. This is consistent with previous studies showing that MD is associated with other psychological disorders (Somer et al., 2016a,c; Somer et al., 2017a). Specifically, this finding is supported by a recent multi-country study by Somer et al. (2020) that reported high levels of depression and anxiety symptoms among probable MDers during the COVID-19 lockdown. Interestingly, mild-to-severe levels of anxiety and depression were equally associated with MDers' mental distress. This finding is in line with previous reports showing that MD can become a dysfunctional coping strategy to avoid negative affect, such as anxiety and depression even if not of such a level as to be considered frank disorders (Somer, 2002; Somer et al., 2020). Hence, to lower the risk of exacerbating their disorder during such adverse situations as the COVID-19 outbreak, it may be important for MDers to gain an awareness of their broader mental condition, particularly when immersion in fantasy is associated with concurrent psychological symptoms.

Surprisingly, negative stress symptoms did not predict MD in our study. This result is in contrast with previous studies that indicated a generally high level of distress in probable MDers compared to non-MDers (Bigelsen et al., 2016). This finding could be partially explained by the fact that during the same period, the Italian population at large reported a high level of distress (Cellini et al., 2020; Moccia et al., 2020; Schimmenti et al., 2020a). Therefore, COVID-19-related contextual factors might have suppressed the significant differences between the two groups of participants.

Interestingly, contrary to our expectations, we found that stable or increased face-to-face relationships and a decreased frequency of online contacts during the COVID-19 lockdown were positively linked with MD. These findings can be interpreted in view of the peculiar characteristics of the pandemic circumstances. During data collection, social relationships in the physical world could represent a potential source of contagion and, therefore, a source of anxiety, whereas online relationships constitute a safe place to engage in meaningful ties with others (Moore and March, 2020). Indeed, it could be argued that some individuals who have been more exposed to the risks of contagion may have displayed excessive absorption in their fantasy world to shield their minds from dysregulated internal states (Ferrante et al., 2020). Consequently, a vicious cycle could evolve in which the withdrawal into an inner world, to avoid the worries associated with the external upheaval, may have contributed to further estrangement from online social relationships and support. Hence, to understand maladaptive outcomes during an enforced social distancing period such as the COVID-19 lockdown, it is important to distinguish between different dimensions of social experience (i.e., face-to-face vs. online social relationships) allowing us to understand the effects that relational variables can have on MD. Consistent with our findings, Somer et al. (2019b, p. 104) already found that the MD acts as a “protective bubble” for some MDers who utilize it to isolate themselves from the external world (Bigelsen and Schupak, 2011). Moreover, the Italian study by Schimmenti et al. (2020c) suggested that detachment, characterized by withdrawal from other people and avoidance of intimate relationships, was a relevant personality feature of MDers. Thus, individuals with probable MD who display this personality feature may have been dissuaded from safely maintaining online social relationships during the COVID-19 lockdown.

As with every research, the present study comes with several limitations. Although our sample size was large, we again acknowledge that our findings are not representative of the entire Italian population. Two main reasons prevent generalizability: first, we employed a convenience snowball rather than a representative method of sampling. We have therefore not defined a priori a minimum number of administrations but only the period useful for the compilation. In fact, our sample included mostly young female adults who are more likely to participate in online surveys (Dillman, 2000). Furthermore, at the time of data collection, a large majority of participants (65.5%) lived in northern Italy, which was a geographical area much more affected by the health emergency than the rest of Italy. This could have led us to overestimate the detrimental psychological effects of the COVID-19 lockdown among the general population in Italy. Second, readers should consider the possibility of potential false positives in our data associated with the screening procedure we adopted. In this study, we used a measure empirically known for its good sensitivity and specificity (Schimmenti et al., 2020c). However, we did not administer the diagnostic “gold standard” structured clinical interview for MD proposed by Somer et al. (2017b). However, the urgent need to complete our data collection in this unfunded study during the time-limited lockdown prevented the interviewing of 1,082 participants.

Additionally, the utilization of self-report measures may have contributed to a response bias. Nevertheless, the tools we used have previously displayed good psychometric properties in worldwide research. Furthermore, due to the cross-sectional nature of the study design, causal inferences cannot be made. Specifically, MD changes during the COVID-19 lockdown were not assessed due to the unpredictable nature of this event and budget limitations. Moreover, retrospective self-reported diagnoses about MDers' condition before the COVID-19 lockdown were not included in this study because they were deemed to be too biased in several studies, especially for mood and anxiety disorders (e.g., Lobato et al., 2012). Hence, we were unable to determine the specific impact of this context on MD levels. Moreover, our cross-sectional data do not allow us to determine whether MD was a dysfunctional coping strategy already used before the COVID-19 lockdown. Longitudinal studies are needed to confirm our findings and to unravel any effects attributable to environmental factors or individuals' pre-existing psychopathology. Specifically, future studies should include a non-lockdown in-depth assessment of MDers to longitudinally evaluate the specific role played by social restriction measures on their psychological functioning.

Notwithstanding these drawbacks, our cross-sectional findings revealed that beyond the distressing effect of the COVID-19 lockdown on the general population, vulnerable individuals, such as probable MDers, may have suffered from psychiatric symptoms that have probably gone beyond the predictable annoyance and distress to be expected in a community sample. Specifically, concerns about contagion or infecting others during the COVID-19 outbreak might exacerbate the tendency of MDers to withdraw into their inner worlds, worsening their mental state and estranging themselves even further from available online social support. Consequently, our findings imply the potential usefulness of Internet-based support platforms for individuals with MD during times of crisis that may require self-isolation. Such emergencies may include natural and environmental disasters, war, and terrorism. These kinds of online platforms should aim at establishing and promoting important coping resources such as enhanced relational security and connectedness. Moreover, from a clinical point of view, we believe that prevention and tailored interventions for MDers should take into account the relationship between social isolation, depression, and anxiety, variables that we suggest are potential triggers and facilitators of the disorder.

## Data Availability Statement

The raw data supporting the conclusions of this article will be made available by the authors, without undue reservation.

## Ethics Statement

The studies involving human participants were reviewed and approved by Ethics Committee of the Center for Research and Psychological Intervention (CERIP). The patients/participants provided their written informed consent to participate in this study.

## Author Contributions

AM and CF provided substantial contributions to the conception of the work, deep analysis of the literature, study design, development, and final approval of the manuscript. LP contributed to data analysis and agreement for final approval of the manuscript. MF, ES, EV, CZ, MQ, VL, GM, DL, PC, LB, RC, GP, and GC contributed to the revision of the work and agreement for final approval of the manuscript. ES and AS contributed to the development and deep revision of the work, with literature analysis and agreement for final approval of the manuscript. All authors contributed to the article and approved the submitted version.

## Conflict of Interest

The authors declare that the research was conducted in the absence of any commercial or financial relationships that could be construed as a potential conflict of interest.
