# Supplementary material for: Maladaptive Daydreaming in an Adult Italian Population During the COVID-19 Lockdown
Source: Front Psychol. 2021 Mar 24;12:631979. doi: 10.3389/fpsyg.2021.631979 (PMC8024516; doi:10.3389/fpsyg.2021.631979)
Supplement: Supplementary file 1 [file Table_1.DOCX]

**Supplementary Table S1.** MDS-16 items

| 1. Some people notice that certain music can trigger their daydreaming. To what extent does music activate your daydreaming? 1. Alcune persone notano che una musica particolare può dare il via ai loro sogni a occhi aperti. In che misura la musica può attivare il tuo sognare a occhi aperti? |
| --- |
| 2. Some people feel a need to continue a daydream that was interrupted by a real-world event at a later point. When a real-world event has interrupted one of your daydreams, how strong was your need or urge to return to that daydream as soon as possible? 2. Alcune persone sentono il bisogno di continuare un sogno a occhi aperti che è iniziato, ma è stato interrotto dopo da un avvenimento del mondo reale. Quando un evento del mondo reale ha interrotto uno dei tuoi sogni a occhi aperti, quanto forte è stata la tua urgenza o il tuo bisogno di tornare a quel sogno il più presto possibile? |
| 3. How often are your current daydreams accompanied by vocal noises or facial expressions (e.g., laughing, talking, or mouthing the words)? 3. Quanto spesso i sogni a occhi aperti che fai sono accompagnati da espressioni vocali o facciali (riso, parole o farfugliamento)? |
| 4. If you go through a period of time when you are not able to daydream as much as usual due to real- world obligations, how distressed are you by your inability to find time to daydream? 4. Se attraversi un periodo di tempo in cui, a causa di impegni nel mondo reale, non riesci a sognare a occhi aperti per come lo fai di solito, quanto ti senti stressato dall’impossibilità di trovare tempo per sognare a occhi aperti? |
| 5. Some people have the experience of their daydreaming interfering with their daily chores or tasks. How much does your daydreaming interfere with your ability to get basic chores accomplished? 5. Ad alcune persone capita che i loro sogni a occhi aperti interferiscano con la loro capacità di svolgere gli impegni o le loro faccende quotidiane. Quanto il tuo sognare a occhi aperti interferisce con la tua capacità di assolvere ai tuoi impegni principali? |
| 6. Some people feel distressed or concerned about the amount of time they spend daydreaming. How distressed do you currently feel about the amount of time you spend daydreaming? 6. Alcune persone si sentono stressate o preoccupate per il tempo che trascorrono sognando a occhi aperti. Quanto ti senti stressato per il tempo che impieghi sognando a occhi aperti? |
| 7. When you know you have had something important or challenging to pay attention to or finish, how difficult was it for you to stay on task and complete the goal without daydreaming? 7. Quando sai che avevi qualcosa d’importante o difficile a cui dovevi prestare attenzione o che dovevi finire di fare, quanto è stato difficile per te continuare l’attività e raggiungere l’obiettivo senza sognare a occhi aperti? |
| 8. Some people have the experience of their daydreaming hindering the things that are most important to them. How much do you feel that your daydreaming activities interfere with achieving your overall life goals? 8. Alcune persone avvertono che il loro sognare a occhi aperti li ostacola nelle cose che sono per loro più importanti. Quanto senti che il tuo sognare a occhi aperti interferisce con il raggiungimento dei tuoi obiettivi di vita? |
| 9. Some people experience difficulties in controlling or limiting their daydreaming. How difficult has it been for you to keep your daydreaming under control? 9. Alcune persone trovano difficoltà nel controllare o limitare i loro sogni a occhi aperti. Quanto è stato difficile per te tenere sotto controllo il tuo sognare a occhi aperti? |
| 10. Some people feel annoyed when a real-world event interrupts one of their daydreams. When the real world interrupts one of your daydreams, on average how annoyed do you feel? 10. Alcune persone si infastidiscono quando un avvenimento del mondo reale interrompe i loro sogni a occhi aperti. Quando il mondo reale interrompe uno dei tuoi sogni a occhi aperti, in che percentuale ti senti infastidito? |
| 11. Some people have the experience of their daydreaming interfering with their academic/occupational success or personal achievements. How much does your daydreaming interfere with your academic/occupational success? 11. Alcune persone avvertono che i loro sogni a occhi aperti interferiscono con i loro successi scolastici, accademici o lavorativi o con la loro realizzazione personale. Quanto il tuo sognare a occhi aperti interferisce con il tuo successo scolastico, accademico o lavorativo? |
| 12. Some people would rather daydream than do most other things. To what extent would you rather daydream than engage with other people or participate in social activities or hobbies? 12. Alcune persone preferiscono sognare a occhi aperti piuttosto che fare molte altre cose. In quale misura preferisci sognare a occhi aperti piuttosto che relazionarti con altre persone o partecipare ad attività sociali o hobby? |
| 13. When you first wake up in the morning, how strong has your urge been to immediately start daydreaming? |
| 13. Quando ti alzi al mattino quanto è forte la tua necessità di cominciare immediatamente a sognare a occhi aperti? |
| 14. How often are your current daydreams accompanied by physical activity such as pacing, swinging, or shaking your hands? |
| 14. Quanto spesso i tuoi sogni a occhi aperti sono accompagnati da movimenti fisici, come fare dei passi, dondolare o muovere le mani? |
| 15. Some people love to daydream. While you are daydreaming, to what extent do you find it comforting and/or enjoyable? 15. Alcuni amano sognare a occhi aperti. Mentre sogni a occhi aperti, in che misura senti che questa esperienza è rassicurante e/o appagante? |
